# Color Stability of Single-Shade Resin Composites: A Systematic Review of In Vitro Studies and Clinical Implications

**DOI:** 10.3390/dj14050293

**Published:** 2026-05-12

**Authors:** Mohamed Mohsen, Daniele Pergolini, Elena Bianca Nistor, Sara Habilaj, Guido Migliau, Flaminia Marini Grassetti, Antonella Polimeni, Gaspare Palaia

**Affiliations:** Department of Oral and Maxillofacial Sciences, Sapienza University of Rome, 00161 Rome, Italy; mmohsen3010@gmail.com (M.M.);

**Keywords:** color stability, coloring agents, composite resins, multi-shade composite resins, single-shade composite resins, spectrophotometry

## Abstract

**Background/Objectives**: Modern restorative dentistry increasingly focuses on achieving optimal aesthetic integration while simplifying clinical procedures. Single-shade resin composites were introduced to reduce the complexity of conventional multi-shade layering techniques through enhanced color adjustment potential and structural color technology. However, concerns remain regarding their long-term color stability. The aim of this systematic review was to evaluate the color stability and aesthetic performance of single-shade resin composites compared with conventional multi-shade systems under different staining and aging conditions. **Methods**: This systematic review was conducted according to PRISMA 2020 guidelines and registered in the Open Science Framework. A systematic search was performed in PubMed, Scopus, and Cochrane Library up to 31 October 2025. Studies evaluating color stability using the CIEDE2000 (ΔE_00_) formula after staining and aging procedures were included. Risk of bias was assessed using the RoBDEMAT tool. **Results**: The search identified 169 records, of which 11 in vitro studies met the inclusion criteria. Across the included studies, discoloration increased progressively over time, and chromogenic agents frequently induced ΔE_00_ values exceeding clinical acceptability thresholds. Turmeric and red wine demonstrated the highest staining potential, whereas distilled water and artificial saliva showed minimal color variation. Single-shade composites generally exhibited greater susceptibility to discoloration than multi-shade systems, although variability among materials was observed depending on composition and experimental conditions. **Conclusions**: Single-shade resin composites simplify restorative procedures and provide initial aesthetic integration; however, they appear more susceptible to clinically relevant discoloration under experimental conditions. Further clinical and in vitro investigations are required to confirm these findings.

## 1. Introduction

Chromatic variations in resin-based materials are influenced by both intrinsic factors, such as the chemical composition of the resin matrix and filler particle size, and extrinsic factors, primarily related to the absorption of pigments from dietary sources. In vitro studies have consistently shown that commonly consumed substances such as coffee, tea, red wine, and turmeric represent the main cause of discoloration, as these pigments interact with the polymer matrix and may penetrate its structure [1,2].

Furthermore, research highlights a strong correlation between surface roughness and staining susceptibility; a rough or inadequately polished surface promotes chromogen adhesion and accumulation, whereas a smooth, well-polished surface is essential to minimize discoloration over time [3,4].

In addition, water sorption phenomena can facilitate matrix swelling and enhance pigment diffusion within the material, further contributing to color instability, as materials that absorb water may also uptake staining agents and undergo matrix degradation [5].

Moreover, the degree of polymerization plays a crucial role, as incomplete monomer conversion may increase the susceptibility of the resin matrix to water sorption, solubility and subsequent discoloration [6].

Overall, these mechanisms highlight the multifactorial nature of discoloration in resin-based materials and the challenges associated with maintaining long-term aesthetic stability.

In modern restorative dentistry, achieving a seamless integration between the restoration and the natural tooth structure is a complex task, as a natural dentition is inherently polychromatic. Traditionally, clinicians relied on multi-shade composites, utilizing complex layering techniques and an extensive inventory of hues and opacities to mimic enamel and dentin. However, these procedures are inherently time-consuming, technique-sensitive, and often lead to material waste [7].

To overcome the limitations of traditional systems, the industry introduced single-shade resin composites. These innovative materials leverage high Color Adjustment Potential (CAP), or the chameleon effect, allowing a single mass of material to adapt to a wide range of VITA classical shades [8,9]. While some systems use traditional pigments, others utilize structural color technology [10,11]. By incorporating uniform spherical fillers of approximately 260 nm, these materials reflect specific light wavelengths through optical interference, effectively blending the restoration with the surrounding tooth structure without the need for chemical dyes [12,13].

However, this simplification may come at a cost, as single-shade composites have been reported to exhibit increased susceptibility to discoloration. The same optical and compositional features that enable the chameleon effect may also influence water sorption, surface degradation and pigment uptake [14,15].

To eliminate the subjectivity of visual assessment, objective color measurement is performed using spectrophotometers, which provide quantitative data by converting reflected light into measurable coordinates. The most accurate and sensitive metric currently used is the CIEDE2000 (ΔE_00_) formula (Equation (1)), which aligns more closely with human visual perception than the traditional CIELAB system [16,17].

For clinical interpretations, the literature establishes a perceptibility threshold (PT) at ΔE_00_ = 0.8 and an acceptability threshold (AT) at 1.8, beyond which color change is considered clinically unacceptable [18,19].

(1) $$∆E_{00}=\sqrt{{\left(\frac{∆L^{\prime }}{K_{L}S_{L}}\right)}^{2}+{\left(\frac{∆C^{\prime }}{K_{C}S_{C}}\right)}^{2}+{\left(\frac{∆H^{\prime }}{K_{S}S_{H}}\right)}^{2}+R_{T}\left(\frac{∆C^{\prime }}{K_{C}S_{C}}\right)\left(\frac{∆H^{\prime }}{K_{H}S_{H}}\right)}$$

The CIEDE2000 color difference formula (ΔE_00_). ΔL, ΔC e ΔH represent the differences in lightness, chroma, and hue, respectively, between the initial and final measurements. SL, SC, and SH are the weighting functions used to adjust the total color difference according to the non-linear sensitivity of the human eye. kL, kC and kH are parametric factors (usually set to 1 in dental research) that account for the influence of experimental conditions. Finally, RT is the rotation function, which oversees the interaction between chroma and hue differences in the blue region of the color space.

Current evidence is largely derived from standardized in vitro protocols, which serve as a fundamental predictive model for material behavior. These studies have been instrumental in identifying the chemical and physical vulnerabilities of the resin–filler interface, demonstrating how specific stressors, such as thermal cycling and prolonged immersion in highly chromogenic media, trigger the degradation of structural color.

Despite promising initial aesthetics, current evidence remains fragmented and largely limited to in vitro studies, with inconsistent findings regarding the color stability of single-shade composites under different staining and aging conditions [18,20].

Therefore, this systematic review aims to evaluate the color stability and aesthetic performance of single-shade composites compared with traditional multi-shade systems, by analyzing in vitro factors affecting discoloration and discussing their limitations to better inform clinical practice.

## 2. Materials and Methods

This systematic review was conducted and reported in accordance with the PRISMA 2020 guidelines [21].The completed PRISMA 2020 checklist is provided in the Supplementary Materials (Table S6).

The following research question was defined using the PICO strategy: “How does the color stability of single-shade resin composites compare with that of conventional multi-shade systems when subjected to standardized aging and staining protocols, as measured by ΔE_00_?”.

The systematic review was registered in Open Science Framework “OSF” Registration DOI: https://doi.org/10.17605/OSF.IO/trcfw.

The authors have stated explicitly that there are no conflicts of interest in connection with this article.

### 2.1. Inclusion and Exclusion Criteria

The selection criteria for this systematic review were defined to ensure the inclusion of relevant and methodologically robust studies. Only resin composites explicitly classified as single-shade, universalshade, or chameleon-effect by manufacturers were included, while conventional multi-shade and non-commercial materials were excluded. Eligible studies included in vitro and clinical investigations evaluating color stability using standardized substrates such as natural teeth, artificial teeth, or resin discs. Studies were required to employ aging or staining protocols, including thermocycling, immersion in chromogenic agents, or environmental simulations, and to report objective color measurements using ΔE_00_ (CIEDE2000). Studies with unclear methodologies, subjective assessments, or incomplete data were excluded. Additionally, only studies published in English or in Italian with relevant scientific data, were considered.

Studies were limited to English and Italian languages due to feasibility considerations; however, this may introduce a potential risk of language bias.

This rigorous selection approach ensured that the included evidence was directly applicable to assessing the color stability and aesthetic performance of single-shade composites (Table 1).

### 2.2. Search Strategy

A systematic search was performed across three databases, PubMed, Scopus and Cochrane Library, and was completed on 31 October 2025, with no time restrictions, using keywords and terms related to composite resins, single shades, and color stability in combination with the Boolean operators “AND” and “OR” (Table 2).

Furthermore, a manual search was performed on the citation and reference lists of the included studies to identify the non-recalled publications in the initial database search.

### 2.3. Selection and Screening Process

Two independent reviewers (M.M. and E.B.N.) performed the study screening in two separate stages. Firstly, both titles and abstracts of the resulting studies were screened independently based on the previously mentioned inclusion and exclusion criteria. Secondly, the confirmation of the selected articles for the review was performed through a full-text read. The disagreements between the two reviewers were resolved by a third reviewer (G.P.) who led the arbitration and discussion in both stages.

Inter-rater agreement statistics were not calculated; disagreements were resolved through consensus and third-reviewer arbitration.

The search identified 57 records from PubMed, 92 studies from Scopus and 20 studies from Cochrane Library, resulting in a total of 169 records. A total of 65 duplicate studies were eliminated. Following title and abstract screening, 73 records were excluded. Thirty-one full-text reports were sought for retrieval; four reports were not retrieved, resulting in twenty-seven reports assessed for eligibility. Sixteen were excluded with explicit reasons (lack of relevant aging/staining protocol, absence of ΔE_00_ outcome, or focus on color matching only), resulting in 11 studies included in the final analysis. These exclusions reflect the predefined eligibility criteria and are consistent with standard PRISMA methodology.

Although both in vitro and clinical studies were considered eligible, no clinical studies meeting the inclusion criteria were identified; therefore, all included studies were in vitro.

### 2.4. Data Extraction and Synthesis Strategy

Data extraction was performed independently by two reviewers using a standardized and pilot-tested form. The following variables were collected from each study: study design, sample characteristics, material type (single-shade and comparator), staining agent, exposure duration, aging protocol, outcome measures, and ΔE_00_ values over time. Discrepancies were resolved by discussion or consultation with a third reviewer.

Prior to synthesis, a structured framework was defined a priori to guide data integration. Given the heterogeneity of the included studies, a narrative synthesis approach was adopted. Studies were systematically organized according to predefined analytical domains reflecting the main determinants of color stability: (i) staining agent (e.g., coffee, red wine, turmeric), (ii) exposure duration, (iii) material type and composition, (iv) comparator category (single-shade vs. multi-shade), and (v) clinical interpretation based on ΔE_00_ thresholds (perceptibility and acceptability).

Within this framework, findings were grouped and compared across studies to identify consistent patterns, including staining hierarchy, time-dependent effects, and differences between material categories. Where available, quantitative data (ΔE_00_ values) were interpreted in relation to clinical thresholds rather than pooled statistically, due to methodological heterogeneity.

This domain-based synthesis ensured a transparent, structured, and reproducible integration of the evidence.

### 2.5. Assessment of Quality and Bias

The assessment of the quality and risk of bias (RoB) was conducted using the RoBDEMAT (Risk of Bias tool for Dental Materials Studies) tool [22].

Each selected study was subjected to the RoBDEMAT tool and scored independently by the same two reviewers (M.M. and E.B.N.), and conflicts were resolved through consensus or arbitration by a third reviewer (G.P.).

The assessment scores were classified into high and low risk; the category “Unclear risk” was assigned when insufficient information was available to assess bias.

The overall risk-of-bias judgment for each study was derived from domain-level assessments following the RoBDEMAT framework. Studies were classified as having low risk when most domains were rated as low-risk, moderate risk when at least one key domain presented concerns, and unclear risk when insufficient information prevented reliable assessment. This approach ensured a transparent and reproducible synthesis of domain-level judgments.

### 2.6. Synthesis Strategy and Meta-Analytic Feasibility

Prior to data synthesis, the feasibility of quantitative pooling was formally assessed. Potential subgroup analyses were explored based on staining agent, exposure duration, comparator type, and outcome metric (ΔE_00_). However, quantitative synthesis was not feasible due to substantial heterogeneity in experimental protocols, including differences in staining media, exposure durations, and aging procedures, which produce non-comparable outcomes.

In addition, most studies reported multiple non-independent measurements (e.g., multiple staining agents and time points within the same specimens), preventing the identification of a single comparable effect size per study. Subgroup analysis was further limited by the small number of studies per category and by inconsistent reporting of statistical parameters, particularly the absence of standard deviations in some cases.

Therefore, a structured narrative synthesis was performed, organizing findings according to predefined analytical domains, including staining agent, exposure conditions, material type, comparator, and clinical thresholds (ΔE_00_ relative to perceptibility and acceptability levels).

## 3. Results

Results are presented according to predefined analytical domains, including staining agent, exposure duration, material type, comparator, and clinical thresholds, to ensure a structured and reproducible synthesis.

### 3.1. Search Results and Study Selection

The initial search covered the period from 2019 to 2025 and identified 57 records from PubMed, 92 from Scopus, and 20 from the Cochrane Library databases.

**Figure 1:**
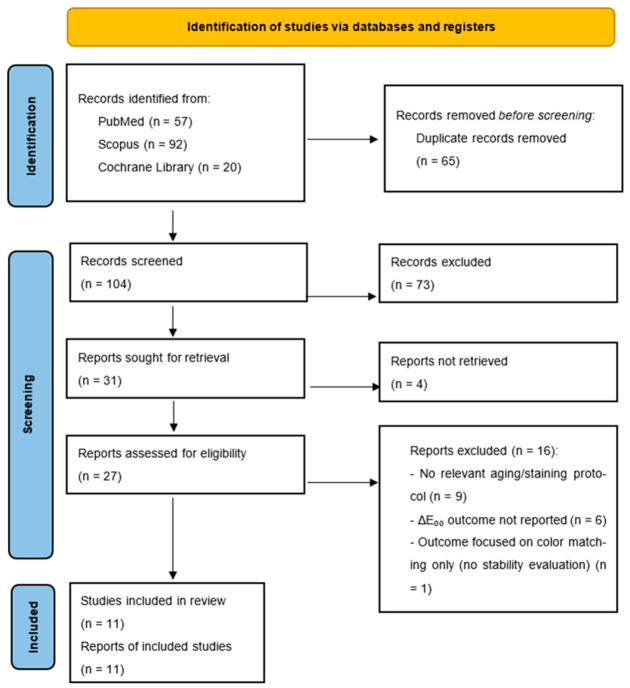
PRISMA 2020 flow diagram of the study selection process.

After removal of 65 duplicates, 104 records were screened based on title and abstract, of which 73 were excluded. Thirty-one reports were sought for retrieval, of which four were not retrieved. Twenty-seven full-text articles were assessed for eligibility. Sixteen were excluded with explicit reasons (lack of relevant aging/staining protocol, absence of ΔE_00_ outcome, or focus on color matching only). Finally, 11 studies were included in the qualitative synthesis (Figure 1). The main characteristics and evaluation criteria of the included studies are summarized in Table 3.

### 3.2. Study Characteristics

All the included studies were in vitro investigations conducted on standardized composite resin samples, discs or blocks made in the laboratory. All included studies evaluated single-shade materials; eight performed a direct comparison with multi-shade composites or, in some cases, with group-shade materials [20,24,25], while the remaining three studies compared different single-shade systems [19,28,29]. The most frequently tested material was Omnichroma^®^, which was evaluated in 6 out of the 11 included studies, often in combination with other single-shade composites such as Vittra APS Unique^®^, ZenChroma^®^, and Charisma Topaz One^®^.

**Figure 2:**
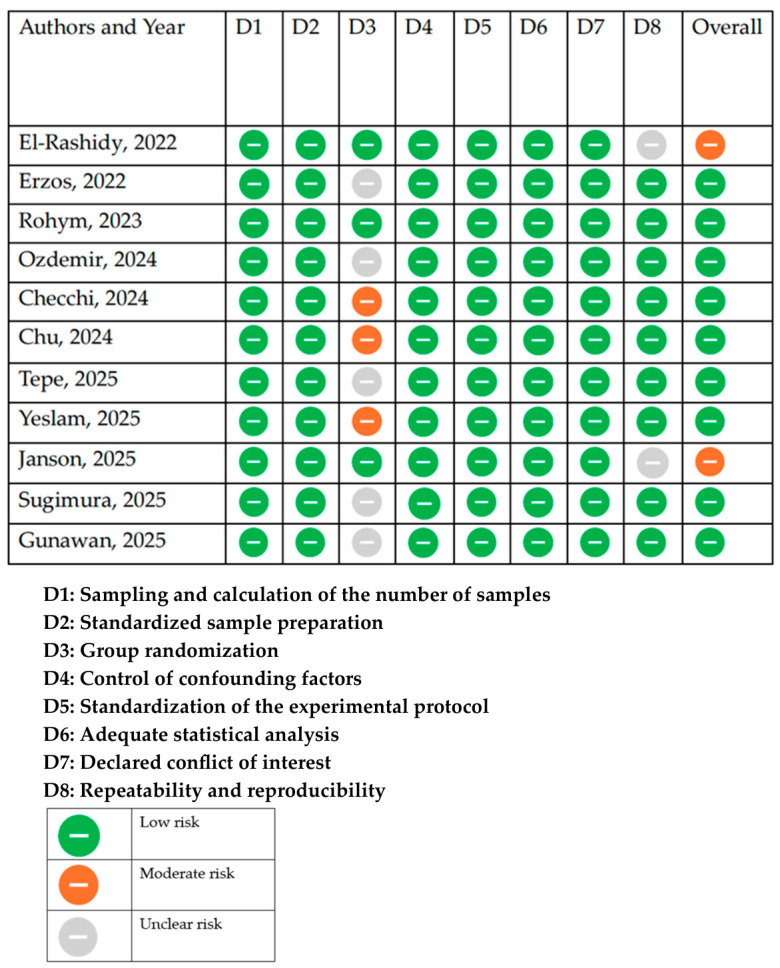
Quality and risk of bias assessment scores using the RoBDEMAT tool of the included studies [18,19,20,23,24,25,26,27,28,29,30].

The GRADE approach was not applied, as it is primarily intended to assess the certainty of evidence in clinical studies with patient-centered outcomes. Since the present review exclusively included in vitro dental materials studies, the RoBDEMAT tool was selected as a more appropriate and validated method for assessing methodological quality and risk of bias in pre-clinical research. However, it should be noted that RoBDEMAT does not provide a formal assessment of the certainty of evidence for clinical decision-making. Therefore, the overall certainty of the evidence supporting clinical implications in this review remains ungraded and should be interpreted with caution. The risk of bias assessment of in vitro studies revealed low risk in nine studies and moderate risk in two studies (Figure 2).

### 3.3. Methodological Characteristics

Spectrophotometry was the predominant method for color assessment (10/11 of the included studies), confirming its role as the reference standard for objective color evaluation [18,29,30].

The ΔE_00_ parameter was consistently used as the primary outcome measure. The color perceptibility and clinical acceptability thresholds, ΔE_00_ = 0.8 (PT, perceptible threshold) and ΔE_00_ = 1.8 (AT, acceptable threshold), were frequently applied to interpret the clinical relevance of color changes [25].

The studies focused on the spectrophotometric evaluation of chromatic variations, primarily using the CIEDE2000 formula, and, in some cases, also the CIE1976 formula for the calculation of the ΔE_00_ and ΔE, respectively.

In one study, the measurements were integrated with digital photographic techniques (CP), which allow the chromatic parameters (L*, a*, b*) to be analyzed using processing software for a comparative visual evaluation (10). Another study used the eLAB digital photographic system, which, like digital photographic techniques (CP), is based on the CIELAB color space, where the L*, a*, and b* coordinates are extracted from the calibrated images to objectively quantify chromatic differences [28].

Spectrophotometry was consistently described as the reference method for color assessment due to its higher sensitivity compared to mobile photography with cross-polarized light [20]. However, methodological parameters, including measurement conditions and calibration protocols, were reported to influence color stability outcomes [26].

Regarding aging and exposure protocols, immersion in staining solutions was the most frequently applied method. Coffee was the most frequently used agent (in 8 out of 11 studies), followed by red wine and tea (black or matcha), while distilled water or artificial saliva served as control conditions. Additional protocols included thermocycling, bleaching procedures, and immersion in highly chromogenic substances such as turmeric, kombucha, and soy sauce [23,26,27].

### 3.4. Color Stability Outcomes

Overall, color variation (ΔE_00_) was influenced by the type of staining agent, exposure time, material composition and aging protocol.

Across the included studies, staining conditions were consistently associated with ΔE_00_ values exceeding the clinical acceptability threshold (1.8), whereas control conditions (distilled water or artificial saliva) predominantly resulted in values below or near this threshold. Time-dependent increases in discoloration were consistently observed, and differences between single-shade and multi-shade composites were reported, although not always consistently across all studies.

Discoloration increased over time, with ΔE_00_ values progressively rising with longer exposure durations [18,19,28]. More intense staining solutions consistently produced ΔE_00_ values exceeding the clinical acceptability thresholds [23,24,28].

Single-shade composites generally exhibited greater color variation compared to multi-shade materials [18,24,25].

The use of ΔE_00_ enabled consistent comparison across studies, although the type of aging medium, material composition, and experimental parameters influenced the observed outcomes.

#### 3.4.1. Exposure Type

Across all included studies, chromogenic solutions consistently induced clinically unacceptable discoloration.

Among staining agents, turmeric and red wine showed the highest discoloration potential. Turmeric, evaluated in 2 of the 11 included studies, produced extreme color changes, with ΔE_00_ values exceeding 16 and reaching approximately 23.91 in single-shade composites after 18 days of immersion [18]. Red wine, assessed in 4 studies, also resulted in high ΔE_00_ values across materials, confirming its strong pigmenting effect.

Coffee, the most frequently used staining agent (in 8 of the 11 included studies), consistently produced ΔE_00_ values exceeding both perceptibility (PT = 0.8) and acceptability thresholds. In some studies, single-shade composites exhibited up to 50% greater discoloration compared to multi-shade materials [27].

Matcha tea demonstrated a staining potential comparable to coffee, with ΔE_00_ values reaching approximately 21–22 after 28 days of immersion [28]. Black tea and kombucha showed moderate staining effects, although still exceeding clinical acceptability thresholds in several cases. In contrast, artificial saliva and distilled water resulted in minimal color variation, with ΔE_00_ values typically around 1.1–1.4, remaining within or close to clinically acceptable limits.

Overall, all chromogenic solutions led to clinically unacceptable discoloration, whereas control conditions produced negligible changes.

#### 3.4.2. Time-Dependent Effects

A consistent time-dependent pattern was observed. The increase in discoloration following the increase in time is clear in the analysis with repeated measures at time 0 (baseline) and after 6, 12, and 18 days of [18], after 7, 14, 21, and 28 days of [28], in the comparison at 1 vs. 14 days of [19], and in the sequence 1–7–30 days of [24].

Short immersion exposures (1–7 days) were sufficient to produce clinically perceptible color changes, with ΔE_00_ values often exceeding acceptability thresholds in strong staining media such as coffee. Prolonged exposure (14–30 days) resulted in values exceeding clinical acceptability thresholds across most staining conditions.

These findings indicate a time-dependent accumulation of discoloration, with ΔE_00_ values increasing progressively with longer exposure durations.

#### 3.4.3. Single-Shade vs. Multi-Shade

In studies including both material categories, single-shade composites generally exhibited higher ΔE_00_ values compared to multi-shade systems under comparable experimental conditions [18,23,25].

This trend was consistently observed across different staining protocols, with single-shade materials showing greater susceptibility to discoloration. In some cases, ΔE_00_ values were up to 50% higher than those recorded for multi-shade composites under the same conditions [27].

For example, Vittra APS Unique^®^ showed higher color change (ΔE_00_ ≈ 23.91) compared to Tetric N-Ceram^®^ (ΔE_00_ ≈ 20.52) after 18 days of immersion. Similarly, ONEshade^®^ demonstrated greater discoloration than OlicoXP^®^ [26].

However, this pattern was not universal. One study reported that Omnichroma^®^ demonstrated better color stability than a multi-shade composite (Filtek Supreme XTE^®^) following thermocycling in staining solutions, indicating that performance may vary depending on material formulation [30].

Variability among different single-shade materials was evident across the included studies, suggesting that color stability is strongly influenced by material composition and formulation rather than by shade category alone [29].

#### 3.4.4. Experimental Conditions

Different aging protocols significantly influenced color stability outcomes across studies. Static immersion in chromogenic solutions, applied in 7 of the 11 included studies, consistently produced the highest ΔE_00_ values, particularly when exposure was prolonged and solutions were renewed daily. This protocol promotes continuous pigment diffusion and water sorption, leading to cumulative discoloration [23,27].

Thermocycling was applied in 4 of the 11 included studies and, when used alone, generally resulted in moderate color changes, consistently exceeding the perceptibility threshold (ΔE_00_ > 0.8) and, in several cases, approaching or slightly exceeding the clinical acceptability threshold. However, these values remained lower than those observed under direct immersion in chromogenic solutions across studies.

Studies employing combined aging protocols (e.g., staining, thermocycling, and brushing simulation) demonstrated more complex and variable outcomes. In these models, mechanical abrasion from brushing may partially reduce superficial staining while simultaneously increasing surface roughness, thereby facilitating further pigment retention. The net effect depended on the balance between these opposing mechanisms [20].

In control conditions, such as distilled water, values remain within the acceptable range and do not induce clinically relevant changes [24,27].

When bleaching is introduced, the impact on the color of single shades is limited: in [29], most materials showed no significant color changes (ΔE*ab and ΔE_00_ ≤ 1.2), with the exception of BU (Beautiful Unishade^®^), which showed a slight increase in ΔE_00_ in the third bleaching session, but still below the thresholds for clinically relevant alteration. Pigmenting conditions lead to values above AT [24,25], while control conditions often fall below or around perceptibility [24,27,28]. Methodologically, the calculation of color change is mainly based on ΔE_00_; some studies also report ΔE*ab alongside ΔE_00_ [18,29].

## 4. Discussion

The present review synthesizes in vitro evidence on the color stability of single-shade resin composites compared with multi-shade systems. Discoloration increased progressively over time across all included studies, chromogenic solutions frequently induced ΔE_00_ values exceeding clinical acceptability thresholds (AT = 1.8), single-shade composites generally showed greater susceptibility to color change, and the magnitude of discoloration varied substantially depending on staining conditions and aging protocols.

Single-shade composites offer clear clinical advantages in terms of simplified workflow and reduced technique sensitivity. However, these benefits appear to be associated with increased susceptibility to discoloration. Across multiple studies, single-shade systems exhibited higher ΔE_00_ values than multi-shade composites under comparable conditions, although variability was observed depending on material formulation. These findings suggest a trade-off between optical adaptability and long-term color stability.

### 4.1. Mechanisms of Discoloration

The variability in ΔE_00_ outcomes reflects the interaction between staining conditions, material composition, and aging protocols. Higher ΔE_00_ values were consistently observed following exposure to highly chromogenic media such as turmeric, red wine, and coffee, indicating strong pigment–material interactions.

This effect was more pronounced in single-shade composites, which showed higher ΔE_00_ values in several studies. Surface degradation further contributed to discoloration: studies using acidic media or combined aging protocols reported greater color change, consistent with increased roughness and pigment retention [31].

A clear time-dependent increase in ΔE_00_ was observed across studies, indicating cumulative discoloration. Prolonged exposure resulted in progressively higher values, often exceeding clinical acceptability thresholds.

Reductions in translucency reported in some studies may explain the loss of color matching after staining and aging. While single-shade composites initially exhibit higher translucency, multi-shade systems generally showed more stable optical behavior under experimental conditions [32,33].

### 4.2. Staining Agents: Clinical Relevance

A consistent hierarchy in staining potential was observed across studies. Turmeric and red wine were associated with the highest ΔE_00_ values, frequently exceeding clinical acceptability thresholds, whereas coffee, tea, and kombucha produced moderate to high discoloration depending on exposure duration.

Turmeric consistently showed the highest ΔE_00_ values, indicating strong staining potential under experimental conditions. Red wine also produced high discoloration, likely reflecting combined staining and acidic effects. Coffee demonstrated reproducible and clinically relevant staining, while tea and kombucha showed cumulative effects over time [34,35].

Control conditions (distilled water or artificial saliva) consistently resulted in minimal color variation, supporting the predominant role of exogenous chromogenic exposure.

Despite these trends, substantial variability was observed across studies, even under similar staining conditions, highlighting the influence of experimental design and material differences.

### 4.3. Material-Related Factors

Material composition significantly influenced discoloration outcomes. Differences in resin matrix and filler characteristics contributed to variability in ΔE_00_ values across studies. Single-shade composites generally exhibited greater susceptibility to discoloration, although this was not consistent across all materials.

These findings indicate that color stability cannot be generalized across composite categories and must be interpreted in relation to both material formulation and experimental conditions.

### 4.4. Degree of Conversion and Photoinitiator Systems

Polymerization efficiency influences resistance to discoloration. Incomplete conversion may increase susceptibility to color change, as reported in several studies [36]. Photoinitiator systems also appear to affect color stability, with some materials demonstrating improved resistance compared with others [37,38].

However, variability across studies suggests that these effects are context-dependent and influenced by experimental design and material composition.

### 4.5. Clinical Implications and Management

From a clinical perspective, single-shade composites simplify restorative procedures but show increased susceptibility to discoloration. In patients with frequent exposure to chromogenic substances, multi-shade composites may provide more stable aesthetic outcomes; however, this inference is based on in vitro evidence and should be interpreted with caution in clinical contexts.

Regardless of material selection, proper finishing and polishing, regular maintenance, and patient education remain essential to minimize discoloration over time [39,40]. Bleaching procedures may reduce superficial staining but do not fully restore original color and may introduce mismatch with natural dentition [41,42].

The absence of eligible clinical studies highlights that all clinical implications in this review are derived from in vitro evidence and should therefore be considered preliminary, requiring confirmation through well-designed clinical investigations.

Methodological heterogeneity across studies further limits comparability. Variability in staining protocols, aging procedures, and material characteristics resulted in non-comparable outcomes. Most studies also reported multiple non-independent measurements, precluding the identification of a single comparable effect size and preventing quantitative synthesis.

Greater variability in ΔE_00_ values was associated with domains reflecting limited control of confounding factors (D4) and insufficient standardization of experimental protocols (D5), which directly informed the overall RoBDEMAT judgments. These methodological aspects are particularly relevant given the sensitivity of in vitro studies to experimental conditions.

This review is limited by the exclusive inclusion of in vitro studies and the absence of a formal inter-rater agreement statistic. Future research should focus on standardized methodologies and long-term clinical studies to improve the reliability and clinical applicability of the evidence.

## 5. Conclusions

Single-shade resin composites simplify restorative procedures and provide effective initial aesthetic integration.

However, compared with conventional multi-shade systems, they appear more susceptible to clinically relevant discoloration, particularly after exposure to chromogenic agents such as red wine, coffee, and turmeric.

Therefore, although single-shade materials may be useful in selected clinical situations, multi-shade systems may offer greater color stability under experimental conditions; however, this conclusion is based exclusively on in vitro evidence and requires confirmation in clinical settings.

Further standardized in vitro studies and well-designed clinical investigations are required to confirm these findings. Therefore, clinical recommendations should be interpreted with caution until supported by longitudinal clinical evidence.

## Figures and Tables

**Table 1:** Inclusion and exclusion criteria.

Domain	Inclusion Criteria	Exclusion Criteria
Type of material	Resin composites explicitly declared as single-shade, universal-shade, or chameleon-effect by the manufacturer (e.g., Omnichroma^®^, Vittra APS Unique^®^, Essentia Universal^®^, Admira Fusion X-tra^®^, Charisma Diamond One^®^, Zenchroma^®^)	Conventional multi-shade composites without color adjustment technology; experimental, prototype, or non-commercial materials
Study design	In vitro and clinical studies evaluating color stability	Case reports, narrative reviews, conference abstracts, letters, non-peer-reviewed publications
Substrate/specimens	Studies using natural teeth, artificial teeth, resin discs, or dentin blocks as substrates for composite evaluation	Studies not clearly specifying the substrate or using non-relevant models
Aging/exposure protocols	Thermocycling, UV exposure, immersion in staining agents (coffee, tea, wine, cola, turmeric, kombucha, etc.), polishing/finishing protocols, bleaching procedures, environmental simulations (pH, humidity, temperature)	Studies without aging, staining, or environmental exposure protocols relevant to discoloration
Outcome measurement	Objective color evaluation using spectrophotometry or calibrated digital systems reporting ΔE_00_ (CIEDE2000)	Studies not reporting ΔE_00_ values or using only subjective/visual color assessment
Data quality	Clearly described, reproducible methodology with verifiable experimental data	Incomplete, unclear, or unverifiable experimental methodology or data
Language	Articles published in English; Italian articles included if containing scientifically relevant data	Articles in other languages
Relevance to topic	Direct evaluation of color stability and aesthetic performance of single-shade composites	Studies focusing on unrelated properties (e.g., only mechanical strength, bond strength, or wear resistance without color evaluation)

**Table 2:** The search and strategy used in the PubMed, Scopus, and Cochrane Library.

Data Base	Search Strategy
PubMed	((“composite resin”[Title/Abstract] OR “resin composite”[Title/Abstract]) AND (“single shade”[Title/Abstract] OR “universal shade”[Title/Abstract] OR “one shade”[Title/Abstract]) AND (“color stability”[Title/Abstract] OR “colour stability”[Title/Abstract] OR “color change”[Title/Abstract] OR “colour change”[Title/Abstract] OR “discoloration”[Title/Abstract] OR “color match”[Title/Abstract] OR “color evaluation”[Title/Abstract] OR “color adaptation”[Title/Abstract] OR “ΔE”[Title/Abstract] OR “ΔE_00_”[Title/Abstract]))
Scopus	TITLE-ABS-KEY (“composite resin” OR “resin composite”) AND TITLE-ABS-KEY (“single shade” OR “universal shade” OR “one shade”) AND TITLE-ABS-KEY (“color stability” OR “colour stability” OR “color change” OR “colour change” OR “discoloration” OR “color match” OR “color evaluation” OR “color adaptation” OR “ΔE” OR “ΔE_00_”)
Cochrane Library	((“composite resin” OR “resin composite”) AND (“single shade” OR “universal shade” OR “one shade”) AND (“color stability” OR “colour stability” OR “color change” OR “colour change” OR “discoloration” OR “color match” OR “color evaluation” OR “color adaptation” OR “ΔE” OR “ΔE_00_”))

**Table 3:** Summarizing and evaluation details of all included studies.

Study (Author, Year)	Study Design	Intervention (Single Shade)	Comparator	Staining & Protocol	Exposure Time	Outcome Measure	ΔE_00_ Range	Exceeds AT (1.8)	Key Findings	Risk of Bias
El-Rashidy et al., 2022 [23]	In vitro	Omnichroma	Filtek Z350 XT	Tea, red wine, thermocycling + immersion	12 days–10,000 cycles	Spectrophotometer	~2.0–15.0	Yes	Greater discoloration in single shade; multi-shade more stable	Low
Ersöz et al., 2022 [24]	In vitro	Omnichroma, Vittra	Multi-shade	Red wine, coffee, tea. Immersion (static, daily renewal)	30 days	VITA Easyshade V	~1.5–12.0	Yes (staining groups)	Higher staining susceptibility, especially with red wine and coffee	Low
Rohym et al., 2023 [19]	In vitro	Omnichroma, Venus Pearl	—	Coffee-immersion (static, daily renewal)	14 days	VITA Easyshade V	~5–26.9	Yes	Significant discoloration and surface roughness increase	Low
Özdemir et al., 2024 [25]	In vitro	Multiple single shade	Neo Spectra ST	Coffee-immersion	24 h	Spectroshade	~3–10.0	Yes	Reduced translucency and increased discoloration; material variability	Low
Checchi et al., 2024 [26]	In vitro	OneShade	Olico XP	Turmeric, soy sauce, energy drink-immersion (daily renewal)	30 days	VITA Easyshade V	~5–20.0	Yes	Turmeric produced highest discoloration	Low
Chen et al., 2024 [27]	In vitro	Charisma Diamond One	Multi-shade	Coffee, thermocycling, bleaching, immersion (static, periodic renewal)	12 days–10,000 cycles	VITA Easyshade V	~1–8.0	Yes (except controls)	Aging increased discoloration; bleaching ineffective	Moderate
Tepe et al., 2025 [20]	In vitro	Multiple single shade	Filtek Z550	Thermocycling, brushing, staining-coffee	10 days/ 1-year simulations	Spectrophotometer + CP	~2–10.0	Yes	All materials showed discoloration; spectrophotometry more reliable	Low
Yeslam & Bakhsh, 2025 [18]	In vitro	Vittra APS Unique	Tetric N-Ceram	Turmeric, kombucha, coffee-immersion (static, staining model)	18 days	Color-Eye 7000A	~7–23.9	Yes	Extreme staining with turmeric; higher discoloration in single shade	Low
Janson et al., 2025 [28]	In vitro	Single-shade systems	—	Coffee, red wine, matcha-immersion (dynamic replacement)	28 days	eLAB system	~1.4–38.9	Yes	Severe discoloration with red wine and matcha; time-dependent increase	Low
Sugimura et al., 2025 [29]	In vitro	Multiple single shade	—	Bleaching	After 3 whitening cycles	Spectrophotometer	≤1.2	No	Bleaching caused minimal color change	Moderate
Gunawan et al., 2025 [30]	In vitro	Omnichroma	Multi-/group-shade	Coffee, red wine-Thermocycling + immersion	10,000 cycles	VITA Easyshade V	~1–17.0	Yes (staining groups)	Some single-shade materials showed comparable stability depending on formulation	Low

## Data Availability

The original contributions presented in this study are included in the article and Supplementary Materials. Further inquiries can be directed to the corresponding author.

## Supplementary Materials

The following supporting information can be downloaded at: https://www.mdpi.com/article/10.3390/dj14050293/s1. Supplementary Table S1: Detailed dataset and synthesis table of included studies; Supplementary Table S2: Full-text excluded studies with individual reasons for exclusion; Supplementary Table S3: Detailed data extraction and cross-study comparison of included studies; Supplementary Table S4: Meta-analytic feasibility assessment and rationale for not performing quantitative synthesis; Supplementary Figure S1 and Supplementary Table S5: Maximum reported ΔE_00_ values (worst-case scenarios) across included studies presented for illustrative purposes only; Supplementary Table S6: PRISMA 2020 checklist.
