# Supplementary material for: Color Stability of Single-Shade Resin Composites: A Systematic Review of In Vitro Studies and Clinical Implications
Source: Dent J (Basel). 2026 May 12;14(5):293. doi: 10.3390/dj14050293 (PMC13205133; doi:10.3390/dj14050293)
Supplement: Supplementary file 1 [file dentistry-14-00293-s001.zip › Supplementary figure S1.pdf]

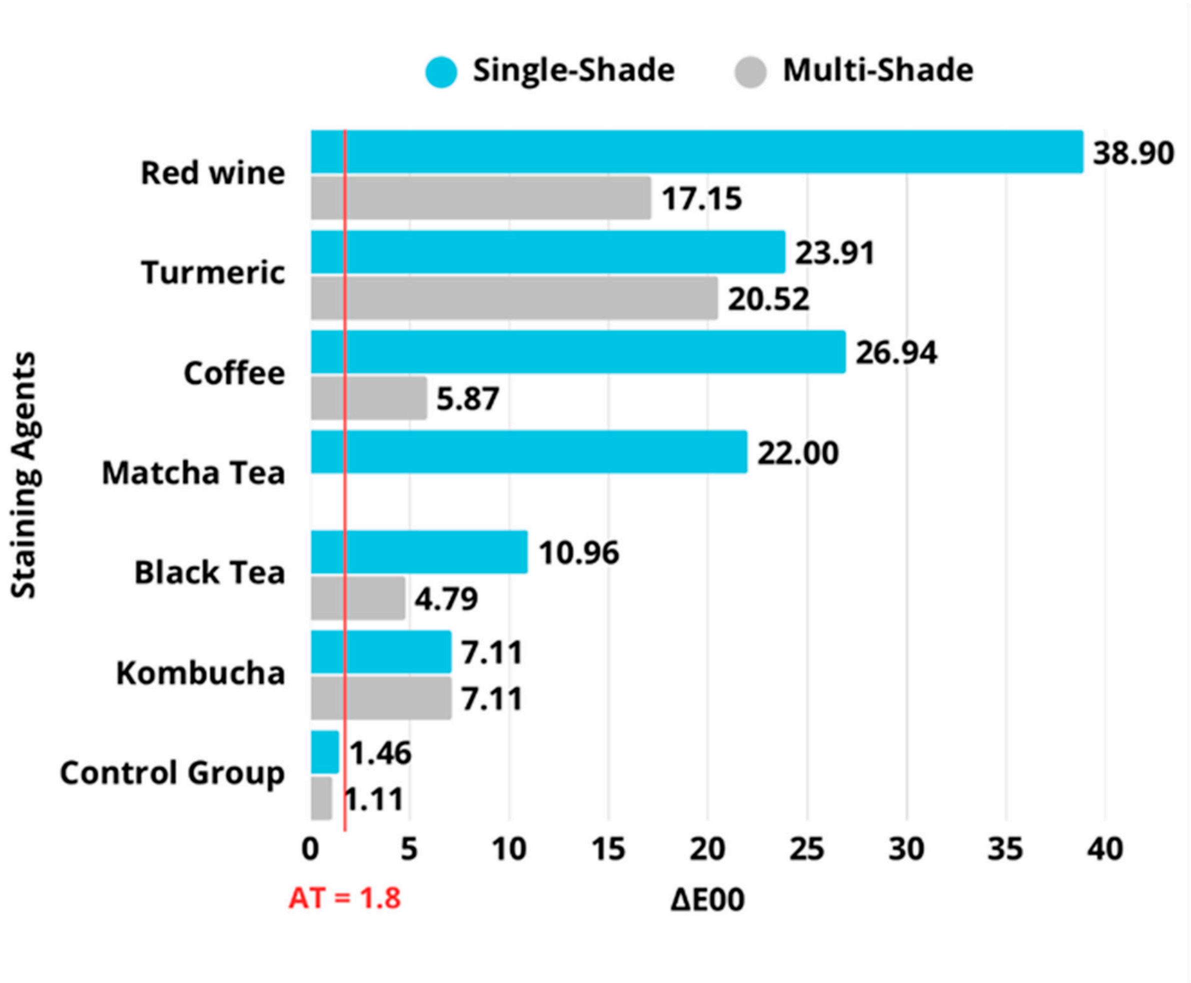


***Supplementary figure S1.*** Comparative illustration of maximum reported color change ($\Delta E_{00}$) values across staining agents. These values represent worst-case outcomes reported in the included studies and are presented for illustrative purposes only. They do not reflect pooled estimates or average effects and should be interpreted in the context of study heterogeneity and experimental conditions *(**Supplementary Table S5**)*.
