# Supplementary material for: Color Stability of Single-Shade Resin Composites: A Systematic Review of In Vitro Studies and Clinical Implications
Source: Dent J (Basel). 2026 May 12;14(5):293. doi: 10.3390/dj14050293 (PMC13205133; doi:10.3390/dj14050293)
Supplement: Supplementary file 1 [file dentistry-14-00293-s001.zip › Supplementary table S2.pdf]

Study (Author, Year)	Reason for Exclusion
de Abreu, J.L.B. et al. (2021)	Lack of relevant aging/staining protocol
Barros, M.S. et al. (2022)	Lack of relevant aging/staining protocol
Nagi, S.M. et al. (2022)	Lack of relevant aging/staining protocol
Truong, T.A. et al. (2023)	Lack of relevant aging/staining protocol
de Livi, G.J.S. et al. (2023)	Lack of relevant aging/staining protocol
Islam, M.S. et al. (2023)	Lack of relevant aging/staining protocol
Bisharah, W.F. et al. (2024)	Lack of relevant aging/staining protocol
Zhu, J. et al. (2025)	Lack of relevant aging/staining protocol
Koi, K. et al. (2025)	Lack of relevant aging/staining protocol
AlHamdan, E.M et al. (2021)	No ΔE00 outcome
Ebaya, M.M et al. (2021)	No ΔE00 outcome
AlHabdan, A et al. (2022)	No ΔE00 outcome
Vejendla, I. et al. (2023)	No ΔE00 outcome
Ozaslan, S. et al. (2024)	No ΔE00 outcome
Karadağ, G. et al. (2024)	No ΔE00 outcome
Ramos, L.V. et al. (2025)	Outcome focused only on color matching
