# Supplementary material for: Color Stability of Single-Shade Resin Composites: A Systematic Review of In Vitro Studies and Clinical Implications
Source: Dent J (Basel). 2026 May 12;14(5):293. doi: 10.3390/dj14050293 (PMC13205133; doi:10.3390/dj14050293)
Supplement: Supplementary file 1 [file dentistry-14-00293-s001.zip › Supplementary table S3.pdf]

Study	Material	Comparator	Staining agent	Exposure time	Aging protocol	ΔE_{00}(mean ± SD)	Exceeds AT	Measurement method	Risk of bias
Ersöz (2022)	Omnichroma (SS)	Multi-shade systems	Red wine	30 days	Immersion (static, daily renewal, 37°C)	15.30 ± 0.9	Yes	VITA Easyshade V	Low
Ersöz (2022)	Omnichroma (SS)	Multi-shade systems	Tea	30 days	Immersion (static, daily renewal, 37°C)	10.72 ± 0.5	Yes	VITA Easyshade V	Low
Ersöz (2022)	Omnichroma (SS)	Multi-shade systems	Coffee	30 days	Immersion (static, daily renewal, 37°C)	8.49 ± 0.3	Yes	VITA Easyshade V	Low
Ersöz (2022)	Omnichroma (SS)	Multi-shade systems	Distilled water	30 days	Immersion (static, daily renewal, 37°C)	1.18 ± 0.2	No	VITA Easyshade V	Low
Ersöz (2022)	Vittra APS Unique (SS)	Multi-shade systems	Red wine	30 days	Immersion (static, daily renewal, 37°C)	17.57 ± 0.9	Yes	VITA Easyshade V	Low
Ersöz (2022)	Vittra APS Unique (SS)	Multi-shade systems	Tea	30 days	Immersion (static, daily renewal, 37°C)	10.96 ± 0.5	Yes	VITA Easyshade V	Low
Ersöz (2022)	Vittra APS Unique (SS)	Multi-shade systems	Coffee	30 days	Immersion (static, daily renewal, 37°C)	8.76 ± 0.8	Yes	VITA Easyshade V	Low
Ersöz (2022)	Vittra APS Unique (SS)	Multi-shade systems	Distilled water	30 days	Immersion (static, daily renewal, 37°C)	1.42 ± 0.3	No	VITA Easyshade V	Low
Ersöz (2022)	Clearfil Majesty ES-2 Premium (MS)	\	Red wine	30 days	Immersion (static, daily renewal, 37°C)	12.56 ± 0.3	Yes	VITA Easyshade V	Low
Ersöz (2022)	Clearfil Majesty ES-2 Premium (MS)	\	Tea	30 days	Immersion (static, daily renewal, 37°C)	6.30 ± 0.8	Yes	VITA Easyshade V	Low
Ersöz (2022)	Clearfil Majesty ES-2 Premium (MS)	\	Coffee	30 days	Immersion (static, daily renewal, 37°C)	4.30 ± 0.2	Yes	VITA Easyshade V	Low
Ersöz (2022)	Clearfil Majesty ES-2 Premium (MS)	\	Distilled water	30 days	Immersion (static, daily renewal, 37°C)	1.12 ± 0.3	No	VITA Easyshade V	Low

Ersöz (2022)	G-aenial A'Chord (MS)	\	Red wine	30 days	Immersion (static, daily renewal, 37°C)	11.24 ± 0.7	Yes	VITA Easyshade V	Low
Ersöz (2022)	G-aenial A'Chord (MS)	\	Tea	30 days	Immersion (static, daily renewal, 37°C)	4.79 ± 0.7	Yes	VITA Easyshade V	Low
Ersöz (2022)	G-aenial A'Chord (MS)	\	Coffee	30 days	Immersion (static, daily renewal, 37°C)	2.80 ± 0.4	Yes	VITA Easyshade V	Low
Ersöz (2022)	G-aenial A'Chord (MS)	\	Distilled water	30 days	Immersion (static, daily renewal, 37°C)	1.11 ± 0.2	No	VITA Easyshade V	Low
Tepe (2024)	Omnichroma (SS)	Filtek Z350 (MS)	Coffee	10 days simulation	Immersion + brushing + thermocycling	3.22 ± 1.34	Yes	Spectrophotometer + CP	Low
Tepe (2024)	Omnichroma (SS)	Filtek Z350 (MS)	Coffee	1-year simulation	Immersion + brushing + thermocycling	9.62 ± 1.39	Yes	Spectrophotometer + CP	Low
Tepe (2024)	Vittra APS Unique (SS)	Filtek Z350 (MS)	Coffee	10 days simulation	Immersion + brushing + thermocycling	7.06 ± 2.18	Yes	Spectrophotometer + CP	Low
Tepe (2024)	Vittra APS Unique (SS)	Filtek Z350 (MS)	Coffee	1-year simulation	Immersion + brushing + thermocycling	8.2 ± 1.72	Yes	Spectrophotometer + CP	Low
Tepe (2024)	Zenchroma (SS)	Filtek Z350 (MS)	Coffee	10 days simulation	Immersion + brushing + thermocycling	2.87 ± 1.15	Yes	Spectrophotometer + CP	Low
Tepe (2024)	Zenchroma (SS)	Filtek Z350 (MS)	Coffee	1-year simulation	Immersion + brushing + thermocycling	5.64 ± 1.96	Yes	Spectrophotometer + CP	Low
Tepe (2024)	Charisma Diamond One (SS)	Filtek Z350 (MS)	Coffee	10 days simulation	Immersion + brushing + thermocycling	7.71 ± 2.04	Yes	Spectrophotometer + CP	Low
Tepe (2024)	Charisma Diamond One (SS)	Filtek Z350 (MS)	Coffee	1-year simulation	Immersion + brushing + thermocycling	9.68 ± 1.38	Yes	Spectrophotometer + CP	Low

Tepe (2024)	Essentia Universal (SS)	Filtek Z350 (MS)	Coffee	10 days simulation	Immersion + brushing + thermocycling	7.01 ± 1.99	Yes	Spectrophotometer + CP	Low
Tepe (2024)	Essentia Universal (SS)	Filtek Z350 (MS)	Coffee	1-year simulation	Immersion + brushing + thermocycling	8.4 ± 1.81	Yes	Spectrophotometer + CP	Low
Tepe (2024)	Filtek Z350 (MS)	\	Coffee	10 days simulation	Immersion + brushing + thermocycling	2.85 ± 0.86	Yes	Spectrophotometer + CP	Low
Tepe (2024)	Filtek Z350 (MS)	\	Coffee	1-year simulation	Immersion + brushing + thermocycling	5.87 ± 1.41	Yes	Spectrophotometer + CP	Low
Chen (2024)	Charisma Diamond One (SS)	Multi-shade systems	Distilled water	12 days	Immersion (static, periodic renewal)	1.26 ± 0.46	No	VITA Easyshade V	Moderate
Chen (2024)	Charisma Diamond One (SS)	Multi-shade systems	Coffee	12 days	Immersion (static, periodic renewal)	3.60 ± 0.66	Yes	VITA Easyshade V	Moderate
Chen (2024)	Charisma Diamond One (SS)	Multi-shade systems	\	10,000 cycles	Thermocycling	3.57 ± 0.69	Yes	VITA Easyshade V	Moderate
Chen (2024)	Tetric N-Ceram (MS)	\	Distilled water	12 days	Immersion (static, periodic renewal)	0.36 ± 0.13	No	VITA Easyshade V	Moderate
Chen (2024)	Tetric N-Ceram (MS)	\	Coffee	12 days	Immersion (static, periodic renewal)	2.19 ± 0.22	Yes	VITA Easyshade V	Moderate
Chen (2024)	Tetric N-Ceram (MS)	\	\	10,000 cycles	Thermocycling	0.74 ± 0.26	No	VITA Easyshade V	Moderate
Chen (2024)	Filtek Z350 XT (MS)	\	Distilled water	12 days	Immersion (static, periodic renewal)	1.12 ± 0.46	No	VITA Easyshade V	Moderate
Chen (2024)	Filtek Z350 XT (MS)	\	Coffee	12 days	Immersion (static, periodic renewal)	3.06 ± 0.25	Yes	VITA Easyshade V	Moderate

Chen (2024)	Filtek Z350 XT (MS)	\	\	10,000 cycles	Thermocycling	0.57 ± 0.22	No	VITA Easyshade V	Mode rate
Chen (2024)	Clearfil Majesty Posterior (MS)	\	Distilled water	12 days	Immersion (static, periodic renewal)	0.56 ± 0.27	No	VITA Easyshade V	Mode rate
Chen (2024)	Clearfil Majesty Posterior (MS)	\	Coffee	12 days	Immersion (static, periodic renewal)	1.93 ± 0.22	Yes (border line)	VITA Easyshade V	Mode rate
Chen (2024)	Clearfil Majesty Posterior (MS)	\	\	10,000 cycles	Thermocycling	0.70 ± 0.20	No	VITA Easyshade V	Mode rate
Özdemir 2024	Omnichroma (SS)	Neo Spectra ST (MS)	Coffee	24 hours	Immersion	2.25 ± 0.36	Yes	Spectroshad e	Low
Özdemir 2024	Zenchroma (SS)	Neo Spectra ST (MS)	Coffee	24 hours	Immersion	1.81 ± 0.33	Yes	Spectroshad e	Low
Özdemir 2024	Charisma Diamond One (SS)	Neo Spectra ST (MS)	Coffee	24 hours	Immersion	2.0 ± 0.67	Yes	Spectroshad e	Low
Özdemir 2024	Vittra APS Unique (SS)	Neo Spectra ST (MS)	Coffee	24 hours	Immersion	2.76 ± 0.84	Yes	Spectroshad e	Low
Özdemir 2024	Neo Spectra ST (MS)	\	Coffee	24 hours	Immersion	1.2 ± 0.17	No	Spectroshad e	Low
Checchi 2025	ONEshade (ONE30) (SS)	OlicoXP (OXP30) (MS)	Artificial saliva	30 days	Immersion (daily renewal, 37°C)	1.3 ± 1.6	No	VITA Easyshade V	Low
Checchi 2025	ONEshade (ONE30) (SS)	OlicoXP (OXP30) (MS)	Turmeric	30 days	Immersion (daily renewal, 37°C)	16.0 ± 1.3	Yes	VITA Easyshade V	Low
Checchi 2025	ONEshade (ONE30) (SS)	OlicoXP (OXP30) (MS)	Energy drink	30 days	Immersion (daily renewal, 37°C)	2.8 ± 0.9	Yes	VITA Easyshade V	Low
Checchi 2025	ONEshade (ONE30) (SS)	OlicoXP (OXP30) (MS)	Soy	30 days	Immersion (daily renewal, 37°C)	7.6 ± 1.1	Yes	VITA Easyshade V	Low

Checchi 2025	OlicoXP (OXP30) (MS)	\	Artificial saliva	30 days	Immersion (daily renewal, 37°C)	0.9 ± 0.3	No	VITA Easyshade V	Low
Checchi 2025	OlicoXP (OXP30) (MS)	\	Turmeric	30 days	Immersion (daily renewal, 37°C)	12.1 ± 0.1	Yes	VITA Easyshade V	Low
Checchi 2025	OlicoXP (OXP30) (MS)	\	Energy drink	30 days	Immersion (daily renewal, 37°C)	1.9 ± 0.5	Yes	VITA Easyshade V	Low
Checchi 2025	OlicoXP (OXP30) (MS)	\	Soy	30 days	Immersion (daily renewal, 37°C)	5.3 ± 1.2	Yes	VITA Easyshade V	Low
Yeslam 2025	Vittra APS Unique (SS)	Tetric N-Ceram (MS)	Coffee	18 days	Immersion (static staining model)	10.22 ± 2.25	Yes	Color-Eye 7000A	Low
Yeslam 2025	Vittra APS Unique (SS)	Tetric N-Ceram (MS)	Kombucha	18 days	Immersion (static staining model)	7.11 ± 2.41	Yes	Color-Eye 7000A	Low
Yeslam 2025	Vittra APS Unique (SS)	Tetric N-Ceram (MS)	Turmeric	18 days	Immersion (static staining model)	23.91 ± 0.40	Yes	Color-Eye 7000A	Low
Yeslam 2025	Tetric N-Ceram (MS)	\	Coffee	18 days	Immersion (static staining model)	8.50 ± 4.07	Yes	Color-Eye 7000A	Low
Yeslam 2025	Tetric N-Ceram (MS)	\	Kombucha	18 days	Immersion (static staining model)	7.11 ± 2.86	Yes	Color-Eye 7000A	Low
Yeslam 2025	Tetric N-Ceram (MS)	\	Turmeric	18 days	Immersion (static staining model)	20.52 ± 0.66	Yes	Color-Eye 7000A	Low
Janson 2025	Clearfil Majesty ES-2 Universal (SS)	\	Coffee	28 days	Immersion (dynamic replacement)	21.8 ± 0.95	Yes	eLAB system	Low
Janson 2025	Clearfil Majesty ES-2 Universal (SS)	\	Red wine	28 days	Immersion (dynamic replacement)	26.7 ± 0.7	Yes	eLAB system	Low
Janson 2025	Clearfil Majesty ES-2 Universal (SS)	\	Matcha	28 days	Immersion (dynamic replacement)	22.0 ± 0.97	Yes	eLAB system	Low
Janson 2025	Clearfil Majesty ES-2 Universal (SS)	\	Saliva	28 days	Immersion (dynamic replacement)	1.46 ± 0.42	No	eLAB system	Low

Janson 2025	Transcend Universal Composite (SS)	\	Coffee	28 days	Immersion (dynamic replacement)	20.2 ± 1.15	Yes	eLAB system	Low
Janson 2025	Transcend Universal Composite (SS)	\	Red wine	28 days	Immersion (dynamic replacement)	35.2 ± 1.45	Yes	eLAB system	Low
Janson 2025	Transcend Universal Composite (SS)	\	Matcha	28 days	Immersion (dynamic replacement)	21.4 ± 1.77	Yes	eLAB system	Low
Janson 2025	Transcend Universal Composite (SS)	\	Saliva	28 days	Immersion (dynamic replacement)	1.26 ± 0.16	No	eLAB system	Low
Janson 2025	Ecosite One (SS)	\	Coffee	28 days	Immersion (dynamic replacement)	22.3 ± 1.51	Yes	eLAB system	Low
Janson 2025	Ecosite One (SS)	\	Red wine	28 days	Immersion (dynamic replacement)	38.9 ± 1.56	Yes	eLAB system	Low
Janson 2025	Ecosite One (SS)	\	Matcha	28 days	Immersion (dynamic replacement)	21.1 ± 0.8	Yes	eLAB system	Low
Janson 2025	Ecosite One (SS)	\	Saliva	28 days	Immersion (dynamic replacement)	1.73 ± 0.21	No (border line)	eLAB system	Low
Sugimura 2025	A・Uno Universal Basic (SS)	\	Bleaching	After 3 whitening cycles	35% H_2O_2	0.51 ± 0.41	No	Spectrophotometer	Moderate
Sugimura 2025	Beautifil Unishade (SS)	\	Bleaching	After 3 whitening cycles	35% H_2O_2	1.24 ± 0.38	No	Spectrophotometer	Moderate

Sugimura 2025	Omnichroma (SS)	\	Bleaching	After 3 whitening cycles	35% H_2O_2	0.75 ± 0.34	No	Spectrophotometer	Moderate
Sugimura 2025	Transcend Universal Composite (SS)	\	Bleaching	After 3 whitening cycles	35% H_2O_2	0.58 ± 0.33	No	Spectrophotometer	Moderate
Gunawan 2025	Single-shade	Multi-shade	Saliva	10,000 cycles	Thermocycling + immersion	2.29 ± 0.31	Yes	VITA Easyshade V	Low
Gunawan 2025	Single-shade	Multi-shade	Coffee	10,000 cycles	Thermocycling + immersion	2.06 ± 0.08	Yes	VITA Easyshade V	Low
Gunawan 2025	Single-shade	Multi-shade	Red wine	10,000 cycles	Thermocycling + immersion	1.06 ± 0.12	No	VITA Easyshade V	Low
Rohym 2025	Omnichroma (SS)	\	Coffee	14 days	Immersion (static, daily renewal)	16.69 ± 3.52	Yes	VITA Easyshade V	Low
Rohym 2025	Omnichroma (SS)	\	Distilled water	14 days	Immersion (static, daily renewal)	2.91 ± 1.30	Yes	VITA Easyshade V	Low
Rohym 2025	Venus Pearl (SS)	\	Coffee	14 days	Immersion (static, daily renewal)	26.94 ± 6.21	Yes	VITA Easyshade V	Low
Rohym 2025	Venus Pearl (SS)	\	Distilled water	14 days	Immersion (static, daily renewal)	3.97 ± 0.67	Yes	VITA Easyshade V	Low
El-Rashidy 2025	Omnichroma (SS)	Filtek Z350 XT (MS)	Saliva	12 days	Immersion	0.86 ± 0.07	No (≈PT)	Cary 5000	Low
El-Rashidy 2025	Omnichroma (SS)	Filtek Z350 XT (MS)	Tea	12 days	Immersion	4.5 ± 0.2	Yes	Cary 5000	Low
El-Rashidy 2025	Omnichroma (SS)	Filtek Z350 XT (MS)	Red wine	12 days	Immersion	8.12 ± 0.29	Yes	Cary 5000	Low
El-Rashidy 2025	Omnichroma (SS)	Filtek Z350 XT (MS)	Tea	10,000 cycles	Thermocycling	3.24 ± 0.2	Yes	Cary 5000	Low

El-Rashidy 2025	Omnichroma (SS)	Filtek Z350 XT (MS)	Red wine	10,000 cycles	Thermocycling	4.3 ± 0.26	Yes	Cary 5000	Low
El-Rashidy 2025	Filtek Z350 XT (MS)	\	Saliva	12 days	Immersion	0.88 ± 0.08	No (≈PT)	Cary 5000	Low
El-Rashidy 2025	Filtek Z350 XT (MS)	\	Tea	12 days	Immersion	3.64 ± 0.27	Yes	Cary 5000	Low
El-Rashidy 2025	Filtek Z350 XT (MS)	\	Red wine	12 days	Immersion	6.52 ± 0.22	Yes	Cary 5000	Low
El-Rashidy 2025	Filtek Z350 XT (MS)	\	Tea	10,000 cycles	Thermocycling	3.39 ± 0.19	Yes	Cary 5000	Low
El-Rashidy 2025	Filtek Z350 XT (MS)	\	Red wine	10,000 cycles	Thermocycling	4.4 ± 0.21	Yes	Cary 5000	Low
