# Supplementary material for: Color Stability of Single-Shade Resin Composites: A Systematic Review of In Vitro Studies and Clinical Implications
Source: Dent J (Basel). 2026 May 12;14(5):293. doi: 10.3390/dj14050293 (PMC13205133; doi:10.3390/dj14050293)
Supplement: Supplementary file 1 [file dentistry-14-00293-s001.zip › Supplementary table S4.pdf]

Candidate subgroup	n studies (n comparisons)	Outcome	SD available	Unit-of-analysis issues	Reason no pooling
Single-shade vs multi-shade comparison	8 studies (~70–80 comparisons)	ΔE_{00}	Yes (majority)	Multiple materials, staining agents, and timepoints per study; shared comparators	No single independent effect size per study; high clinical and methodological heterogeneity
Coffee staining	8 studies (~60 comparisons)	ΔE_{00}	Yes	Repeated measures and multiple materials per study	Exposure times ranged from 24 h to 30 days or simulated aging; non-comparable protocols
Red wine staining	4 studies (~15–20 comparisons)	ΔE_{00}	Yes	Multiple arms within studies	Limited number of studies; heterogeneous immersion and thermocycling conditions
Tea (black tea)	2 studies (~8 comparisons)	ΔE_{00}	Yes	Multiple materials per study	Insufficient number of studies; protocol variability
Matcha tea	1 study (6 comparisons)	ΔE_{00}	Yes	Multiple materials within a single study	Only one study available
Turmeric staining	2 studies (~6 comparisons)	ΔE_{00}	Yes	Multiple arms	Too few studies; heterogeneous exposure duration and protocols
Kombucha staining	1 study (4 comparisons)	ΔE_{00}	Yes	Single study design	Not poolable
Soy sauce / energy drink	1 study (~6 comparisons)	ΔE_{00}	Yes	Single study with multiple conditions	Not poolable
Static immersion protocols	7 studies (~50 comparisons)	ΔE_{00}	Yes	Multiple media and timepoints per study	Differences in solution type, renewal frequency, temperature, and duration
Thermocycling (alone or combined)	4 studies (~20 comparisons)	ΔE_{00}	Partial	Often combined with staining or brushing	Protocols not comparable (cycles, sequence, media)
Combined aging (staining + brushing + thermocycling)	2–3 studies (~15 comparisons)	ΔE_{00}	Yes	Multi-factorial repeated-measures design	Effects not isolable; no comparable effect size
Bleaching procedures	1 study (4 comparisons)	ΔE_{00}	Yes	Single intervention study	Insufficient data
Exposure time variability	11 studies (>100 total timepoint comparisons)	ΔE_{00}	Yes	Multiple non-independent timepoints per study	Timepoints not independent; durations not comparable
