# Supplementary material for: Color Stability of Single-Shade Resin Composites: A Systematic Review of In Vitro Studies and Clinical Implications
Source: Dent J (Basel). 2026 May 12;14(5):293. doi: 10.3390/dj14050293 (PMC13205133; doi:10.3390/dj14050293)
Supplement: Supplementary file 1 [file dentistry-14-00293-s001.zip › Supplementary table S5.pdf]

Study Ref	Staining Agents	Material	Type	ΔE_{00}	Standard Deviation
[24]Ersöz et al. (2022)	Black Tea	Vittra Unique	Single-shade	10.96	0.50
[24] Ersöz et al. (2022)	Black Tea	G-aenial A'Chord	Multi-shade	4.79	0.70
[24]Ersöz et al. (2022)	Control Group	G-aenial A'Chord	Multi-shade	1.11	0.20
[19] Rohym et al. (2023)	Coffee	Venus Pearl	Single-shade	26.94	6.21
[25]Janson et al. (2025)	Red Wine	Ecosite One	Single-shade	38.90	1.56
[25] Janson et al. (2025)	Matcha Tea	Clearfill Majesty ES-2 Universal	Single-shade	22.00	0.97
[25] Janson et al. (2025)	Control Group	Clearfil Majesty ES-2	Single-shade	1.46	0.42
[27] Gunawan et al. (2025)	Red Wine	Filtek Supreme	Multi-shade	17.15	1.47
[20] Tepe et al. (2025)	Coffee	Filtek Z550	Multi-shade	5.87	1.41
[18] Yeslam et al. (2025)	Turmeric	Vittra ASP Unique	Single-shade	23.91	0.40
[18] Yeslam et al. (2025)	Turmeric	Tetric-N-Ceram	Multi-shade	20.52	0.66
[18] Yeslam et al. (2025)	Kombucha	Vittra APS Unique	Single-shade	7.11	2.41
[18]Yeslam et al. (2025)	Kombucha	Tetric N-Ceram	Multi-shade	7.11	2.86

Supplementary Table S5. Maximum reported ΔE_{00} values (worst-case scenarios) across included studies.
