# Supplementary material for: Color Stability of Single-Shade Resin Composites: A Systematic Review of In Vitro Studies and Clinical Implications
Source: Dent J (Basel). 2026 May 12;14(5):293. doi: 10.3390/dj14050293 (PMC13205133; doi:10.3390/dj14050293)
Supplement: Supplementary file 1 [file dentistry-14-00293-s001.zip › Supplementary table S6.pdf]

PRISMA 2020 Checklist

Section and Topic	Item #	Checklist item	Location where item is reported
**TITLE**			
Title	1	Color Stability of Single-Shade Resin Composites: A Systematic Review of In Vitro Studies and Clinical Implications	Pag. 1
**ABSTRACT**			
Abstract	2	Structured abstract reporting background, aim, methods, results and conclusions.	Pag.1
**INTRODUCTION**			
Rationale	3	Traditional multi-shade composites require complex layering procedures, whereas single-shade composites simplify restorative workflows through color adjustment and structural color effects. However, their long-term color stability remains a major concern because discoloration is a common cause of restoration failure.	Pag. 2
Objectives	4	To assess the color stability and aesthetic performance of single-shade composites compared with traditional multi-shade systems, by focusing on factors affecting discoloration, including staining agents, exposure time, material composition and aging protocols, and outlining their limitations to inform clinical practice.	Pag. 3
**METHODS**			
Eligibility criteria	5	Studies were included if they evaluated resin composites explicitly defined as single-shade or universal shade by manufacturers, using standardized substrates (natural teeth, artificial teeth, or resin discs), and assessed color stability through objective measurements (ΔE_{00}) after aging or staining protocols. Both in vitro and clinical studies were considered eligible; however, no clinical studies met the inclusion criteria, and all included studies were in vitro. Exclusion criteria included non-commercial materials, studies without ΔE_{00} outcomes, subjective assessments, incomplete methodologies, and non-peer-reviewed publications.	Pag. 4-5
Information sources	6	Pubmed, Scopus and Cochrane Library were searched up to October 31, 2025.	Pag. 5
Search strategy	7	Full search strategies for each database are reported in Table 2 and Supplementary Material.	Table 2; Supplementary Table S1
Selection process	8	Two independent reviewers (M. M. and E.B.N.) performed the screening of the studies in two separate stages. Firstly, both titles and abstracts of the resulting studies were screened independently based on the previously mentioned inclusion and exclusion criteria. Secondly, the confirmation of the selected articles for the review was performed through a full-text read. The disagreements between the two reviewers were resolved by a third reviewer (G. P.) who led the arbitration and discussion in both stages.	Pag. 6
Data collection process	9	Data extraction was performed independently by two reviewers using a standardized data extraction form. Discrepancies were resolved through discussion or consultation with a third reviewer.	Pag. 6
Data items	10a	Extracted data included study characteristics (authors, year), type of materials, comparator, staining agents, aging protocols, exposure time, and ΔE_{00} values.	Pag.6
	10b	Additional variables included measurement methods (spectrophotometer or digital systems), surface treatments, and threshold interpretation (perceptibility and acceptability).	Pag. 6
Study risk of bias assessment	11	The risk of bias of the included studies was assessed using the RoBDEMAT (Risk of Bias tool for Dental Materials Studies). Each study was independently evaluated by two reviewers, and disagreements were resolved through consensus or consultation with a third reviewer. The tool assesses multiple methodological domains (e.g., sample preparation, randomization, protocol standardization, and statistical analysis), and studies were classified as low, moderate, or risk unknown based on the reported criteria.	Pag. 6
Effect measures	12	The primary effect measure was color change expressed as ΔE_{00} (CIEDE2000), interpreted according to perceptibility (0.8) and acceptability (1.8) thresholds.	Pag. 4-5
Synthesis	13a	Studies eligible for synthesis were identified and grouped according to staining agents, exposure time, material type, and aging protocols.	Pag. 6

PRISMA 2020 Checklist

Section and Topic	Item #	Checklist item	Location where item is reported
methods	13b	Studies were grouped based on staining agents, exposure time, material type, and aging protocols.	Pag. 6
	13c	A qualitative synthesis approach was used to summarize findings across studies.	Pag. 6
	13d	Due to substantial heterogeneity in study design, materials, and outcome reporting, a meta-analysis was not performed.	Pag. 6
	13e	No subgroup or sensitivity analyses were conducted.	Pag. 6
	13f	No statistical software or quantitative synthesis methods were applied.	Pag. 6
Reporting bias assessment	14	Reporting bias was not assessed.	Not assessed
Certainty assessment	15	Certainty of evidence (GRADE) was not applied, as it is designed for clinical studies; instead, risk of bias was assessed using the RoBDEMAT tool.	Not assessed
**RESULTS**			
Study selection	16a	The search identified 57 records from PubMed, 92 from Scopus, and 20 from Cochrane Library, for a total of 169 records. After removing 65 duplicates, 104 records remained. Following title and abstract screening, 73 records were excluded. Thirty-one full-text reports were sought for retrieval; four could not be retrieved, leaving 27 reports assessed for eligibility. Of these,16 were excluded based on predefined criteria. Ultimately, 11 studies were included in the qualitative synthesis.	Pag.6-8; Figure 2; Supplementary material
	16b	Sixteen were excluded with explicit reasons: lack of relevant aging/staining protocol (n=9), absence of ΔE_{00} outcome (n=6), and focus on color matching only (n=1)	Pag.6-8; Figure 2; Supplementary material
Study characteristics	17	Table 3	Table 3
Risk of bias in studies	18	Figure 3	Figure 3
Results of individual studies	19	Table 3	Table 3
Results of syntheses	20a	The results showed that discoloration was influenced by staining agents, exposure time, and material composition. Turmeric, red wine, and coffee produced the highest ΔE_{00} values, frequently exceeding clinical acceptability thresholds. Single-shade composites generally exhibited greater discoloration compared to multi-shade systems.	Pag. 10-14
	20b	No statistical synthesis was performed.	Not performed
	20c	Variability in results was observed due to differences in materials, staining protocols, and exposure durations across studies.	Pag. 10-14
	20d	No subgroup or sensitivity analyses were conducted.	Not performed
Reporting biases	21	Reporting bias was not assessed.	Not assessed
Certainty of evidence	22	Certainty of evidence was not assessed using GRADE.	Not assessed
**DISCUSSION**			
Discussion	23a	Across the included studies, consistent patterns emerged: discoloration increased over time, chromogenic solutions frequently induced ΔE_{00}	pag.14-17

Section and Topic	Item #	Checklist item	Location where item is reported
		values exceeding clinical acceptability thresholds (AT = 1.8), single-shade composites generally showed greater susceptibility to color change, and the magnitude of discoloration varied substantially de-pending on staining conditions and aging protocols.<br>Single-shade composites offer clear clinical advantages in terms of simplified workflow and reduced technique sensitivity. However, these benefits appear to be associated with increased susceptibility to discoloration. Across multiple studies, single-shade systems exhibited higher ΔE_{00} values than multi-shade composites under comparable conditions, although variability was observed depending on material formulation. These findings suggest a trade-off between optical adaptability and long-term color stability.	
	23b	The primary limitation of the included evidence is that all studies are in vitro, which fails to fully replicate the complex oral environment, including biofilm formation, saliva buffering, and continuous pH fluctuations.	Pag.14-17
	23c	The findings should be interpreted with caution due to heterogeneity in study designs, materials, and experimental conditions.	Pag. 14-17
	23d	Clinically, multi-shade systems remain the gold standard for high-aesthetic-risk patients (frequent consumers of turmeric, wine, or coffee). For single-shade restorations, meticulous multi-step polishing and regular professional maintenance are mandatory to minimize pigment attachment. Future research should prioritize long-term in vivo clinical trials.	Pag. 17
**OTHER INFORMATION**			
Registration and protocol	24a	The systematic review was registered in Open Science Framework “OSF” Registration DOI. : https://doi.org/10.17605/OSF.IO/trcfw.	Pag. 3
	24b	The review protocol is available on OSF (DOI provided).	Pag. 3
	24c	No amendments to the registered protocol were reported.	Not reported
Support	25	No specific funding was received for this study.	Pag. 18
Competing interests	26	The authors have stated explicitly that there are no conflicts of interest in connection with this article.	Pag. 18
Availability of data, code and other materials	27	Data supporting the findings are available within the article and supplementary materials.	Pag. 18

From: Page MJ, McKenzie JE, Bossuyt PM, Boutron I, Hoffmann TC, Mulrow CD, et al. The PRISMA 2020 statement: an updated guideline for reporting systematic reviews. BMJ 2021;372:n71. doi: 10.1136/bmj.n71

For more information, visit: http://www.prisma-statement.org/
